# Applying self‐determination theory towards motivational factors of physical activity in people undergoing haemodialyses: A qualitative interview study

**DOI:** 10.1111/hex.13757

**Published:** 2023-04-04

**Authors:** Mei Huang, Honghong Lv, Aili Lv, Feng Yang, Yuning Tang, Yang Li, Yan Hua, Hongbao Liu, Chunping Ni

**Affiliations:** ^1^ Department of Basic Nursing, School of Nursing Air Force Medical University Xi'an Shaanxi China; ^2^ Department of Nephrology Second Affiliated Hospital of Air Force Medical University Xi'an Shaanxi China; ^3^ Nursing Faculty, Health Science Center Xi'an Jiaotong University Xi'an Shaanxi China

**Keywords:** haemodialyses, physical activity, qualitative study, self‐determination theory, semistructured interview

## Abstract

**Introduction:**

The level of physical activity of people undergoing haemodialyses is low, so understanding what factors underlie the motivation to be physically active in people undergoing haemodialyses is important. Therefore, this qualitative study aims to explore the different motivation types and corresponding basic psychological needs (BPNs) of people undergoing haemodialyses based on self‐determination theory.

**Methods:**

We adopted the objective sampling method to select 19 patients with the end‐stage renal disease aged from 28 to 66 years old from a tertiary hospital in Xi'an. They underwent haemodialyses five to six times every 2 weeks for more than 3 months. Then, we conducted semistructured one‐on‐one interviews with 19 people undergoing haemodialyses using qualitative content analysis. All interviews were recorded, transcribed verbatim and analyzed on a thematic analysis.

**Results:**

We analyzed four motivation types of patients, namely four themes, including entrenching in physical inactivity (Amotivation), breaking physical inactivity (Controlled motivation), finding one's way (Autonomous regulation) and enjoying the positive effects of physical activity (Intrinsic motivation). Each motivation is dominated by one or more BPNs. For example, inadequate Competence such as decreased physical function is the reason why the patient does not perform physical activities. Due to the lack of health education on physical activity, people undergoing haemodialyses often lack the motivation for controlled regulation. The motivation for self‐regulation is generated by the patients' promotion of meeting BPNs, such as normal social interactions. The formation of patients' autonomous motivation can't be separated from the effective understanding felt by other patients, because their situations are similar. Enjoying physical activity promotes the formation of patients' intrinsic motivation and the maintenance of this behaviour.

**Conclusion:**

Perceived Competence, Relatedness and Autonomous Motivation are important determinants for physical activity in people undergoing haemodialyses. Patients need to internalize the changed values and skills, so as to generate the motivation of self‐regulation, rather than external or controlled forms of motivation regulation, to better maintain behaviour change.

**Patient or Public Contribution:**

People undergoing haemodialyses were involved in the development of the interview topic guide to ensure all relevant topics were explored.

## 1 INTRODUCTION

Physical activity, including activities and purposeful exercise as part of daily living, decreases as kidney disease progresses, reaching a nadir in people undergoing haemodialyses.^1^ The physical activity of people undergoing haemodialyses is significantly lower than that of healthy people of the same age and sex and shows a progressive decline, only reaching 35% of that of sedentary people without kidney disease.^2^ It is also associated with poor health‐related quality of life and increased mortality.^3^, ^4^, ^5^ Meanwhile, people undergoing haemodialyses have severe symptoms and comorbidities^6^ that can negatively impact physical functioning^7^ and quality of life.^8^, ^9^ This also reduces the patient's physical activity, which in turn deepens the limitations of the patient's physical functions, forming a vicious circle.^10^

Interestingly, physical inactivity is a major modifiable risk factor for poor health‐related quality of life, morbidity and mortality in people undergoing haemodialyses.^11^ People undergoing haemodialyses who are more physically active have been shown to have a lower risk of death compared with sedentary patients.^12^ The UK Kidney association recommends that people undergoing haemodialyses aim for at least 30 min or moderate intensity of physical activity five times a week, and emphasizes that even small increases in physical activity may provide some benefit.^13^

Although the above‐mentioned ‘people undergoing haemodialyses need more physical activity’, this information failed to change the behaviour of people undergoing haemodialyses. To enhance an individual's physical activity, we, therefore, need to understand and explain, so that we can finally intervene in the factors that affect behaviour change. Most of the previous studies used quantitative questionnaires to study the benefits and barriers of physical activity in people undergoing haemodialyses, qualitative research can explore when and how these factors affect physical activity.^14^, ^15^, ^16^ Previous studies based on social cognitive theory only considered the number of motivations (i.e., motivated and not motivated) for patients with chronic kidney disease, and only explored the external factors that generated motivation for physical activity, such as the benefits of physical activity to overall health.^17^, ^18^ All of these studies, without exception, explained the direction of behaviour, but did not explain how such behaviour was motivated.^19^

Self‐determination theory (SDT) is an organismic theory about human motivation. Its premise is that human beings have internal needs, and behaviour is not only a response to information or punishment, which provides a useful framework for better understanding the motivation behind patients' participation in sports activities.^20^, ^21^ The theory holds that individuals actively pursue three basic psychological needs (BPN): Autonomy, Competence and Relatedness. Autonomy refers to the need for individuals to act, control and support behaviours and make decisions. Competence is the need to feel capable and effective when completing tasks. Relatedness is the need to experience meaningful connections with others in one's own environment. When patients participate in physical activity and meet their three BPN, their self‐determined motivation will develop and enhance.^22^ More specifically, patients would enjoy the activity (Intrinsic motivation), integrate it into their lifestyle and realize that physical activity is important because it has related benefits (Autonomous regulation). On the other hand, if the BPN is not satisfied, the patient would get more controlled physical activity or movement. Controlled regulations reflect participation in physical activity with an internal sense of obligation to avoid guilt, or obtain external incentives or avoid punishment. Both Autonomy regulation and Controlled regulations belong to extrinsic motivation. Amotivation implies a lack of motivation and interest in activities. Self‐motivated behaviours are associated with more favourable physical and mental health outcomes.^23^ On the contrary, those social conditions that undermine these psychological needs will lead to more controllable motives, thus causing the pressure of obedience to damage behaviour. SDT emphasizes the importance of motivation quality more than motivation quantity because different motivation types will produce different activity states. And motivation quality is self‐regulated.^24^

The purpose of this study is to explore the different types of motivation and corresponding BPN of patients when they participate in physical activities based on SDT. This will help researchers design future physical activity interventions to promote BPN satisfaction and compliance.

## 2 MATERIALS AND METHODS

### 2.1 Design

A basic qualitative design was used to complete interviews with patients.^25^ Patients were interviewed using individual semistructured interviews suitable for exploring individuals' experiences and opinions.

### 2.2 Study setting and participants

The study was conducted in the haemodialyses ward of a tertiary hospital in Xi'an, China, from March to June 2022. Patients who met the inclusion and exclusion criteria were contacted after a clinical consultation and obtained a patient information form. Participants with end‐stage renal disease, receiving five or six haemodialyses every 2 weeks and more than 3 months met the inclusion criteria for this study. Participants with cognitive impairment who couldn't cooperate with the interviews; and patients with severe physical limitations to activity were excluded. Purposeful sampling methods were then used to ensure that the sample was differentially representative in terms of sex, age and frequency of haemodialyses. All participants who meet the inclusion and exclusion criteria have signed the informed consent form. The researcher obtained the patient information form from the medical staff, so as to contact the patient after the interview. This study was approved by the Ethics Committee of Xi'an Jiaotong University (No. 20211575).

### 2.3 Data collection

All patients were interviewed face‐to‐face in the haemodialyses unit, which lasted 30–45 min. The interview was conducted by M. H., a researcher who systematically studied the relevant theories of qualitative research and repeatedly practiced and confirmed that she has mastered the skills of a qualitative interviews. Interviews followed a semistructured format that was developed based on research objectives, previous research and clinical observations (Supporting Information: Table 1). Before using the interview topic guide, we selected 2–4 people undergoing haemodialyses for preinterview, and further modified and deleted the interview topic guide according to the effect of the preinterview to determine the final version of the interview topic guide. All interviews were recorded digitally, anonymously and verbatim by professionals. Read transcripts while listening to audio files to ensure accuracy. The verified transcript is returned to the patient for examination to ensure accuracy. All audio files and scripts were imported into NVivo 11 (QSR International NVivo 11 Pro). Demographic and clinical data were extracted from medical records.

### 2.4 Data analysis

Data collection and analysis for this study were conducted concurrently to explore new concepts in the remaining interviews. Data were analyzed using qualitative content analysis.^26^ The analysis was performed in collaboration with M. H. and H. L. and validated by M. H. with experience in qualitative research.

The researchers first read the interview record through, read it repeatedly and got a general understanding. Then, researchers encoded interviews using a broad coding scheme (open coding). The code for each interview has been revised and reviewed and divided into categories and subcategories. Emerging categories and subcategories were edited to avoid overlap between categories and excessive heterogeneity within individual categories.^27^ The research team stopped the inclusion of research subjects when no new information appeared, and finally, 19 research subjects participated in the interview, meeting the principle of data saturation.

## 3 RESULTS

Nineteen people undergoing haemodialyses were invited to take part in the study. The demographic characteristics of each participant are illustrated in Table 1.

**Table 1:** Patient characteristics.

Identification for interview	Gender	Age	Haemodialyses vintage	Pathogeny	Number of complications	Haemodialyses frequency
A	Female	31	1.5	Hypertensive nephropathy	1	5 times/2 weeks
B	Female	49	5.3	Hypertensive nephropathy	1	5 times/2 weeks
C	Female	62	3.2	Hypertensive nephropathy	1	5 times/2 weeks
D	Male	62	10.9	Glomerulonephritis	1	3 times/week
E	Male	59	5.3	Hypertensive nephropathy	1	5 times/2 weeks
F	Male	28	4.6	Unknown	0	5 times/2 weeks
G	Male	63	4.0	Diabetes nephropathy	3	5 times/2 weeks
H	Female	40	0.9	Glomerulonephritis	0	3 times/week
I	Female	66	6.1	Unknown	2	5 times/2 weeks
J	Male	28	0.9	Glomerulonephritis	1	3 times/week
K	Female	64	5.1	Diabetes nephropathy	2	5 times/2 weeks
L	Male	57	6.7	Diabetes nephropathy	2	3 times/week
M	Male	44	0.6	Polycystic nephropathy	2	5 times/2 weeks
N	Male	32	0.3	Unknown	0	3 times/week
O	Male	39	1.9	Glomerulonephritis	1	3 times/week
P	Female	37	1.0	Unknown	1	5 times/2 weeks
Q	Male	39	2.3	Diabetes nephropathy	1	5 times/2 weeks
R	Male	60	3.1	Unknown	1	5 times/2 weeks
S	Male	60	4.7	Glomerulonephritis	0	5 times/2 weeks

*Note*: Age and haemodialyses duration expressed in years at the time of interview.

**Figure 1:**
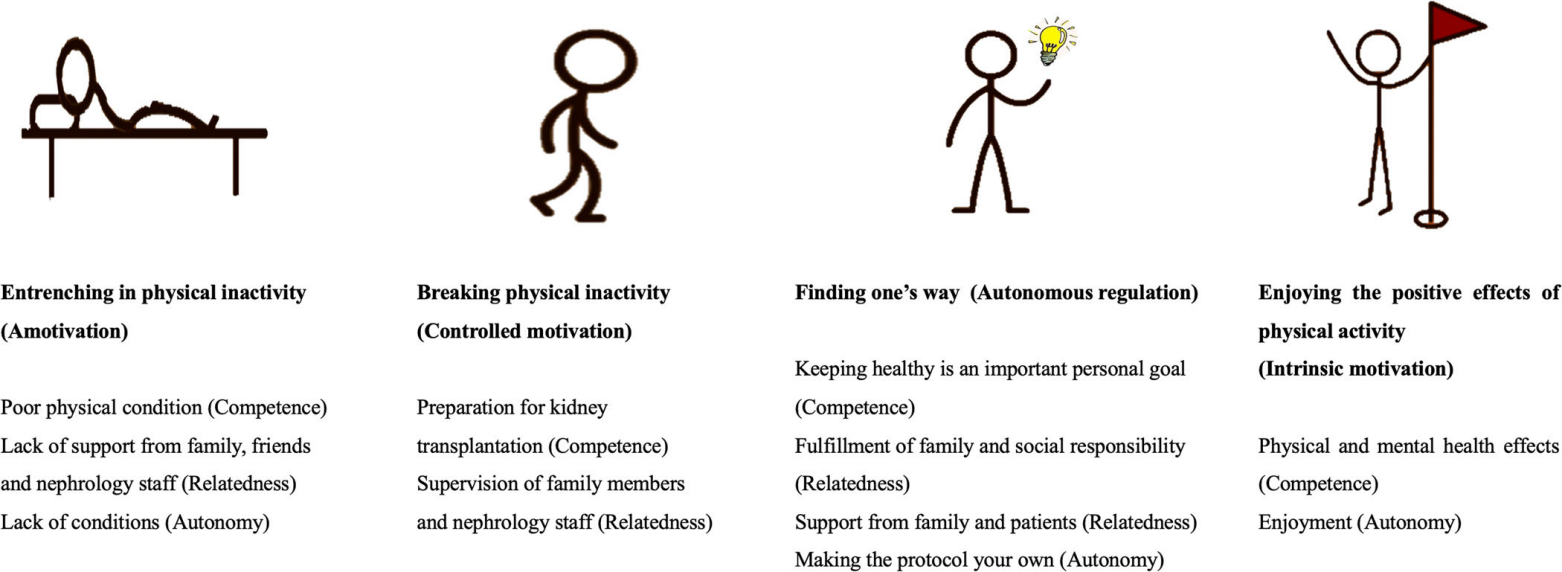
Motivation types and basic psychological needs.

On the basis of main attributes of SDT, the interview results obtained 4 main categories of patients' motivation and 12 subcategories reflecting the BPN (Figure 1). The four main categories are respectively, entrenching in physical inactivity (Amotivation), breaking physical inactivity (Controlled regulation), finding one's way (Autonomous regulation) and enjoying the positive effects of physical activity (Intrinsic motivation). Tables 2, 3, 4, 5 list those with relevant subcategories and example citations.

**Table 2:** Entrenching in physical inactivity (Amotivation) exemplar quotations.

Major themes	Minor themes	Exemplar quotations
Poor physical condition (Competence)	Symptom burden	The muscles may be significantly less. Because I feel that my arms and legs are getting more and more tired, that is, I will have no energy all over my body. (Q1, F)
		I was very tired after hemodialyses, and I just wanted to go home and lie down to rest or sleep. (Q2, R)
	Increasing age and haemodialyses vintage	I feel this way myself, because I have been through it for a long time, um, it will definitely get worse as I go on. (Q3, N)
	Physical activity related concerns	While I may feel that increasing physical activity is no different from everyday life, my body responds. For example, panting, especially sweating a lot, chest tightness and shortness of breath, I feel that my own breath is not enough for myself. (Q4, F)
		Before I started hemodialyses, I swam and played badminton. I never swam after hemodialysis because I was afraid that fistula would be infected by unclean swimming pool. (Q5, D)
	Negative comparison	I used to go to the gym to run on the treadmill, but now I don't dare to run on the treadmill, because sometimes the speed is accidentally increased, and I can't keep up. Then I don't want to do any activities. (Q6, P)
Lack of support from family, friends and nephrology staff (Relatedness)	Lack of support from family	My family told me to stay at home and not to go out and worry about my accident. And I have diabetes, I'm afraid I'm outside low blood sugar, what if something happens? (Q7, M) I've now put a burden on my home. Who helps to watch a grandchild while I'm doing hemodialysis treatment? is it? I had previously fallen down the stairs, tripped the hand, fractured the bone, and played a plaster. My wife said to have spared you from getting this arm of the arteriovenous fistula. (Q8, D)
	Friends estranged	We have a team playing badminton and swimming, and now they don't dare to call me. They are also afraid because I am not a normal person. There's nothing wrong with being inactive anyway, right? (Q9, D)
	Inconsistent health education help from staff	No one said to me to be active. I don't know it (physical activity) and don't value that. (Q10, N) The nurse has said that I had better move about, but she simply said a few words. (Q11, A)
Lack of conditions (Autonomy)	Physical environment	I won't go out for activities when it rains. When it is too cold, I am afraid of catching a cold. (Q12, B)

**Table 3:** Breaking physical inactivity (Controlled motivation) exemplar quotations.

Major themes	Minor themes	Exemplar quotations
Preparation for kidney transplantation (Competence)	Meet the weight requirement of kidney transplantation	After all, there are some weight requirements for transplantation. Being physically active is still good for my weight control. (Q13, H)
Supervision of family members and nephrology staff (Relatedness)	Guilt for family members	Because before I didn't raise dogs, I didn't sleep at night, could not come to bed in the morning and could not sleep normally. You see, when my son asked me to have this dog, he was telling me to go out and do more activities, rather than stay at home all the time. (Q14, B)
	Suggestions of nephrology staff	The doctors and nurses only asked me to control my weight during hemodialysis and try to keep it as long as possible (weight), but they didn't provide specific information about physical activity. (Q15, C) I can walk just, and doctors don't let me run ah. Previously the director said to try to avoid strenuous exercise, do not take a big deal of movement, is to walk a walk, trot down, not too long. Do not race like others because, after all, I have the disease. (Q16, P) I think it's good to walk, not to sleep at home. (Q17, A)

**Table 4:** Finding one's way (Autonomous regulation) exemplar quotations.

Major themes	Minor themes	Exemplar quotations
Keeping healthy is an important personal goal (Competence)	Fear of rapid decline of physical condition	I was afraid that I would not be able to walk. These patients who have come here for more than ten years will be in wheelchairs. What should I do? I am afraid of this….There is a dialysis aunt here, who is over 60 and in good spirits. She always looks after her daughter. She walks two or three kilometers every day, and she deliberately goes to this distant vegetable market to buy vegetables. She has been through it for decades. I think she is in good spirits. (Q18, O) There is a patient who doesn't like to walk and always lies down. She just suffers a lot. (Q19, G)
Fulfillment of family and social responsibility (Relatedness)	Assuming family responsibilities	I have a child over three years old. He basically stays in one place and plays all the time. Well, I'm going to stand there all day. It would be better if there was a seat to sit on. I would feel uncomfortable if I couldn't. Because I'm not very active in general, I can't support taking care of my children. (Q20, N)
	Worry about losing job	I am a teacher. I go out to teach others. If people see me in a very poor state and guess that I am a patient, they will doubt whether my teaching content is correct, right? (Q21, E)
Support from family and patients (Relatedness)	Encouragement and companionship of family	When it rains, my wife always accompanies me for a walk at Wetland Park. (Q22, E)
	Discussing with other patients	I have discussed with my fellow patients, just run three kilometers a day, walk two kilometers, anyway, this day. (Q23, O)
Making the protocol your own (Autonomy)	Stepwise change in personal exploration	My own understanding of staying active may mean moving every day. But you can't exercise vigorously, just do what you can. (Q24, J) I started by walking five kilometers and then I figured it out myself. I added the amount, jogged for three kilometers, started running one kilometer, and could run two kilometers or three kilometers. Now I basically run three kilometers and then walk back, that's it. (Q25, O)
	Integrating into daily life	I may walk about 10,000 steps a day, if I don't come to the hospital. Every time (without hemodialysis), I just walk straight from our company to the subway line 3, and then it is estimated that it will be four or five stops, about 10,000 steps. (Q26, A)
	Using convenient resources effectively	I live on Fangwei Road, very close to the hospital. When I came for hemodialysis and when I left, I didn't take a car, I just walked, and I walked fast. (Q27, G) I am jobless. Anyway, I send my wife to work in the morning, so I go running, which can also pass the time. (Q28, O) I have a WeChat public account, where I can read and download related knowledge about people undergoing hemodialysis, and there is content on how to do activities. Anyway, just look at these (public accounts) information. (Q29, C)

**Table 5:** Enjoying positive effects of physical activity (Intrinsic motivation) exemplar quotations.

Major themes	Minor themes	Exemplar quotations
Physiological and psychological effects (Competence)	Physical function improvement	I think doing regular physical activity is also very powerful. In the past ten years, I have never had this flu, and I have not taken cold medicine once in more than ten years. I think this is an encouragement to me. (Q30, I)
	Full of energy and vitality	I don't have the energy if I don't walk. If I walk more often now, I will feel better. (Q31, L)
	Reduced restrictions on basic needs	I can't drink water if I'm not active. Because as soon as I drink water, the weight will increase, and then the amount of hemodialysis will increase. If the amount of hemodialysis is increased, I will suffer. I walk more every day; I can sweat and drink more water. (Q32, R)
Enjoyment (Autonomy)	Sense of achievement	I think it's still useful to go out and walk around every day. The doctors all said, ‘You see this old lady is still doing well’. I think this sentence gave me a lot of encouragement. (Q33, I)
	Enjoying being active	Last year on National Day, even though it was cold and rainy, I ran. I was running hot, sweating, and feeling very comfortable. Now I enjoy running more and more. (Q34, O) I was physically active every day. Even though I was a little uncomfortable that day and did not want to run, I would still walk, taking a 20 minute, nearly 30 minutes path. (Q35, P)

### 3.1 Entrenching in physical inactivity (Amotivation)

Exemplar quotations for this category are presented in Table 2.

Some participants were reluctant to change due to insufficient Competence, lack of experience of relevance and lack of conditions, so they are usually in a state of physical inactivity.

#### 3.1.1 Poor physical condition (Competence)

Comorbid conditions and symptom burden were described as common features of physical inactivity in people undergoing haemodialyses. Symptoms were considered functional limitations in people undergoing haemodialyses and most commonly included fatigue, pain, and shortness of breath. Besides, patients acknowledged that aging and haemodialyses duration process were unalterable facts and limited the patient's physical function year by year. Middle‐aged and elderly patients feel more strongly. Patients described accidental injuries were caused by activities overestimating their physical ability. Participants also avoided activities involving the arms due to problems such as arteriovenous fistula stenosis. In particular, patients who enjoyed moderate‐to‐high‐intensity activity before haemodialyses had the strongest negative contrast, causing their interest in physical activity to plummet.

#### 3.1.2 Lack of support from family and friends (Relatedness)

Failed physical activity experiences created fear of activity in the patient and, more importantly, were the strong reason for the family to discourage the patient from being active. Going out with friends or colleagues was a good opportunity for patient activity. However, the excessive sense of responsibility of friends was worried that going out will cause secondary harm to the patients, which gradually reduced the opportunities for the patients to participate in activities. At the same time, it also damaged the social interaction of patients, and the concept of ‘patients are sick and different from normal people’ was deeply rooted in the patients' mind.

Although nephrologists regularly see patients, they have poor health education on physical activities for patients. Some patients said that they never learned this from the staff of the nephrology department, and thought that the staff was too busy to explain the physical activity to patients. At the same time, the patient felt that the staff did not mention physical activity, indicating that it was not important.

#### 3.1.3 Lack of external conditions (Autonomy)

Patients described in detail how severe weather hindered their plans to travel outside.

### 3.2 Breaking physical inactivity (Controlled regulation)

The patients dominated by Controlled motivation also began to perform physical activities, but their execution and persistence were poor. Exemplar quotations for this category are presented in Table 3.

#### 3.2.1 In preparation for kidney transplantation (Competence)

Overweight patients usually lose weight through physical activity to successfully obtain additional rewards, that is, to reach the standard of kidney transplantation.

#### 3.2.2 Supervision of family members and staff (Relatedness)

Family members of patients are important factors in the external supervision of patients' physical activities. Undetailed health education also created external supervision for some patients. Some patients said that the staff let them do light physical activity; some reported that doctors do not allow patients to exercise vigorously. As a result, the patient concluded that walking every day is the most suitable activity for them. In addition, when staff asked patients to control their weight, the patients also indirectly knew that they needed to do physical activities.

### 3.3 Finding one's way (Autonomous regulation)

Patients who started Autonomous regulation began to feel their preference for physical activities and could gradually integrate them into their lives. At the same time, patients put forward new requirements for their own BPN and want to meet BPN by changing physical activity. Exemplar quotations for this category are presented in Table 4.

#### 3.3.1 Keeping healthy is an important personal goal (Competence)

It is not surprising that many patients hope to obtain the expected health benefits from physical activity, including feeling healthier, and improving their sense of well‐being and quality of life. Although this form of motivation was initially an external motivation, accompanied by some fears about the consequences of being sedentary, many participants have internalized health threats as the result of changes of personal importance. Often, these motivations are based on participants' desire to avoid painful symptoms experienced by other people undergoing haemodialyses. For example, patients hope to make their legs strong enough through physical activity, slow down the decline of physical functions, be able to walk stably and delay the use of wheelchairs. Patients with poor health are considered as typical examples of a sedentary state, such as yellow or black skin, which is described as ‘visible on the face’. Some patients become active because they are afraid of being the same as the above patients. In addition, prolonging life is the ultimate goal of patients and one of the sources of motivation for patients to start physical activity.

#### 3.3.2 Fulfilment of family and social responsibility (Relatedness)

In addition to improving basic Competence, some patients believed to keep healthy to get along with their families and fulfill their family responsibilities, as well as maintain their normal social roles and functions. Most patients reported that the frequent haemodialyses have brought financial burdens to the family and dragged down the family. A young patient reported that he wanted to maintain a normal family income and raise children, while the older patient believed that improving physical fitness would reduce the extra burden of troublesome children to transport themselves for haemodialyses treatment. Some patients also explained that showing their illness to the patient at the social level would arouse sympathy from others. Therefore, for patients, maintaining a person's normal social function is an important requirement for starting physical activities.

#### 3.3.3 Support from family and patients (Relatedness)

Over time, the family's companionship is an automatous regulation by which the patient can proactively integrate physical activity into life. Meanwhile, patients with similar physical functions spontaneously formed patient circles to encourage each other and discuss their physical activity experiences.

#### 3.3.4 Making the protocol your own (Autonomy)

Participants mentioned how, over time, their lifestyle behaviours were motivated and progressively changed by the formation of a new pattern and routine. Most patients did physical activity according to their physical condition. The common stopping criteria for patients were subjectively ‘tired’ and ‘slight sweating’. The number of steps patients explored for this was typically 5000 to 10,000 steps. Because the time and place for physical activity are flexible, patients can smoothly integrate physical activity into daily life, and can easily achieve the specified goals. For patients who go to work, commuting is the best way to increase physical activity. Walking on haemodialyses and housework were also appropriate methods for patient selection.

Convenient activity resources, such as flexible time and accessibility to facilities, helped patients develop the habit of physical activity. For example, compared with gyms, parks were the most accessible and low‐cost resources for people undergoing haemodialyses. Internet information has become easy access for patients to learn about physical activity.

### 3.4 Enjoying the positive effects of physical activity (Intrinsic motivation)

When patients enjoy physical activity, they will insist on physical activity all the time. Exemplar quotations for this category are presented in Table 5.

#### 3.4.1 Physiological and psychological effects (Competence)

Participants said that their new lifestyle behaviours were pleasant or personally satisfying. Some of them described in detail how they gradually improved their physical condition after insisting on physical activity. Patients reported how physical activity transforms negative emotions into positive ones, making them happier and more energetic. Drinking water is the most basic physiological demand of patients, and it is also the basic life function limitation of haemodialyses. Physical activity helps patients sweat and drink more water, which has become an important way for patients to control their weight besides haemodialyses.

#### 3.4.2 Enjoyment (Autonomy)

The patient's adherence to physical activity promoted the formation of a new lifestyle. The stronger the physical and mental benefits they perceived, the greater their sense of achievement, which encouraged the patient to keep going, creating a virtuous circle. The sense of achievement also came from the improvement of the patient's own comprehensive state, especially when compared with other sedentary patients. The affirmation of professionals made the patient's sense of achievement reach its peak. Enjoyment of physical activity was an important determinant of maintaining physical activity. The patient spoke with pride when it came to running when it rained and going out for a walk with his or her neighbours when it snowed.

## 4 DISCUSSION

According to SDT, the results describe the four motivation types of physical activity and the corresponding BPN of people undergoing haemodialyses. It can be seen that when three BPNs are not satisfied, the patient has no motivation to do physical activities. When the three BPNs are satisfied, the patient will adjust motivation autonomously, which is beneficial to maintain physical activity.

When people undergoing haemodialyses have no motivation for physical activity, it shows that they are in a state of low physical activity. The patient's Competence, Relatedness and Autonomy are unmet in this motivation type. Amongst them, patient's physical function is decreased, that is, Competence, which is insufficient to support patients to participate in physical activity. Patients also use external conditions as an excuse to reduce their Autonomy, such as bad weather.

Nephrology staff are the best candidates to teach physical activity knowledge to people undergoing haemodialyses. However, unlike mass exercise rehabilitation for cardiopulmonary disease, physical activity has not become a healthcare priority for nephrology staff.^28^ This is related to the lack of detailed and clear guidelines.^29^ Moreover, the lack of formal referral channels for the rehabilitation of people undergoing haemodialyses may lead to nonstandard patient counselling provided by nephrology staff.^30^ Specific guideline development has been identified as a future research focus.^31^ For patients, the external motivation, especially the Controlled regulation, usually comes from the patient's compliance with the recommendations or goals of the nephrology staff to avoid negative outcomes (such as decreased physical function). For example, Sebire's research on patients with type 2 diabetes and Stewart's research on patients with chronic obstructive pulmonary disease suggest that patients with chronic diseases may need a certain threshold of controlled motivation. Patients can only make initial behaviour changes under the pressure of controlled motivation.^32^, ^33^ However, at present, the common external motivation of people undergoing haemodialyses is only for the fixed population, that is, people waiting for kidney transplantation, and only to make the body weight reach the standard. This may also be related to the fact that the above‐mentioned patients don't receive health education on physical activity. In SDT, controlled forms of extrinsic motivation sometimes regulate (or stimulate) short‐term behavior, but will not be maintained over time. However, it can make a strong preparation for the patient to change from more controlled motivation to more autonomous motivation.^32^, ^33^

Although most patients do not have a controlled motivation, when patients realize that maintaining good health is an important goal of individuals, they will begin to adjust autonomously.^34^ In addition to worrying about the insufficient competence caused by physical inactivity, patients also worry about their social interaction decline. This mainly comes from the patients' attention to health, quality of life and family responsibilities. They expect physical activities to meet their physical and mental health needs and maintain their family roles and functions. Similar to other chronic diseases, the burden of haemodialyses care often extends beyond the patient's scope to their family members,^35^, ^36^ which is closely related to the meaning of family in traditional Chinese culture. In China, family is generally a serious barrier to patients' physical activity.^37^ In China, caring for sick family members is the responsibility of other family members.^38^ Families do not allow sick family members to do household chores, discourage or even limit the normal activities of patients. Patients are unable to fulfill their family roles and become more vulnerable, and they have to rely on family members.^39^ Therefore, patients feel guilty about their families, not only because their illness has brought an economic burden to their families, but also the burden of human and material resources. Studies have shown that families with people undergoing haemodialyses spend an average of 70 h per week on caring activities^40^ and have a lower quality of life than the general population. Patients are eager to improve physical function through physical activity and reduce the burden on the family through activities such as housework. Moorman's findings suggest that most people undergoing haemodialyses supported exercise at home.^16^

However, patients feel less support from their families and friends. Most patients in our study reported that their family and friends discouraged them from doing physical activities. This is similar to what Stewart et al. found in an interview with a pulmonary rehabilitation patient.^33^ This is because family and friends do not understand the illness and limitations experienced by the patient. The lack of understanding by others means that patients have less meaningful contact with others.^41^ But from another perspective, at the same time, patients choose other patients to meet this basic need. This is related to the high similarity between patients, that is, they can establish contact by understanding each other's experiences. First, patients in similar situations are more likely to support and encourage each other and are more confident in physical activity.^42^ Second, it may improve self‐efficacy to observe that patients with the same attributes successfully complete tasks. In addition to this, comparisons between patients can stimulate peer learning.^42^ Therefore, in terms of physical activity, the social circle of patients has evolved from friends before illness to other people undergoing haemodialyses. Patients enjoy discussing and performing physical activities together. It is suggested to organize group discussions for patients to provide them with opportunities to learn and encourage each other to promote and maintain daily sports activities. In addition, it is worth noting that groups can also be grouped by age, as social interactions with peers provide a sense of security to each other. For example, divide the patients into three groups: young, middle‐aged and elderly. Lange's participants also strongly supported this view.^43^

The reduction of negative life experiences of haemodialyses is the most profound positive impact that patients feel after increasing physical activity. The ability to meet the activities of daily living is an important component of quality of life.^44^ Life participation refers to the ability to participate in daily life events (e.g., work, travel, recreation, and learning) and is a critical outcome for patients on haemodialyses.^45^, ^46^ In Wodskou et al. study, patients emphasized the hope that intradialytic exercise would make patients more active and reduce fatigue, while nurses focused on reducing dietary restrictions.^18^ These benefits were felt by patients in our study. This suggests that physical activity can deliver the functional benefits of structured exercise that patients and nurses expect, and is supported by chronic kidney disease patients' perceptions of physical activity.^47^, ^48^ Future specialty referrals may convey the message that physical activity improves a patient's ability to carry out activities of daily living independently.

It can be seen that the benefits of active physical activity as perceived by the patient under the incentive of intrinsic motivation. More importantly, the sense of achievement is the continuous driving force for patients to persist in physical activity. Research by Meis et al. support this view.^49^ This is because over time, the effects of physical activity are fully realized and the patient gradually enjoys the process, and then the extrinsic motivation becomes more internalized, promoting the maintenance of physical activity.^43^ Moreover, their BPN has been met, and patients are more likely to show initiative during physical activity.^33^ In fact, some people believe that when a person has an intrinsic and determined motivation, the lifestyle behaviour of maintaining physical activity is most likely, and can better cope with the common challenges of behaviour change (such as time shortage, holidays and daily changes). Extensive research showed the importance of self‐motivated physical activity participation forms, because compared with behaviours based on external motivation and less internal motivation, autonomous and voluntary behaviours will bring greater participation and durability.

Although this study provides new insights into the determinants of the transition from physical inactivity to physical activity in people undergoing haemodialyses, there are some limitations. The participants screened in this study may be a group of people undergoing haemodialyses interested in physical activity. The sedentary patient may have refused our interview, and we do not fully understand their lack of motivation. Interestingly, the reasons for the decline in interview participants were also factors that affected physical activity levels. Therefore, the results may not cover all potential barriers. Future research will benefit from capturing more sedentary patients. In addition, we did not have the dynamic change process of physical activity behaviour motivation of patients from receiving haemodialyses treatment, nor did we provide strong evidence for the motivation continuum in SDT.

## 5 CONCLUSION

The results of this study suggest that clinicians should consider the quality of patients' motivation, not just the quantity. BPN plays an important role in the change of patients' motivation. For example, the most basic thing is that patients want to be able to participate in activities of daily living, they also need to receive health education from nephrology staff, and the understanding and support of families can promote their self‐regulation motivation. Moreover, health services should actively support the physical activity of people undergoing haemodialyses and provide regular physical activity counselling. Employees also may need to have the skills to engage (motivational interviewing) and motivate patients through dialogue. As for patients, they need to internalize the changed values and skills, so as to generate the motivation of self‐regulation, rather than external or controlled forms of motivation regulation, to better maintain behaviour change.

## AUTHOR CONTRIBUTIONS

**Mei Huang** and **Honghong Lv**: Conceptualization. **Mei Huang**: Methodology. **Feng Yang**: Software. **Yuning Tang, Yang Li** and **Yan Hua**: Validation. **Aili Lv**: Formal analysis. **Aili Lv**: Investigation. **Mei Huang**: Resources. **Honghong Lv**: Data curation. **Mei Huang**: Writing—original draft preparation. **Chunping Ni**: Writing—review and editing. **Honghong Lv**: Visualization. **Honghong Lv**: Supervision. **Chunping Ni**: Project administration. **Chunping Ni**: Funding acquisition.

## CONFLICT OF INTEREST STATEMENT

The authors declare no conflict of interest.

## ETHICS STATEMENT

The study was conducted in accordance with the Declaration of Helsinki, and approved by the Ethics Committee of Xi'an Jiaotong University (protocol code 2021‐1575 and date of approval 23 November 2021). Informed consent was obtained from all subjects involved in the study.

## Supporting information

Supplementary Table 1. Interview questions for hemodialysis participants.

## Data Availability

The original contributions presented in the study are included in the article/Supporting Information: Material, and further inquiries can be directed to the corresponding authors.
