# Supplementary material for: Applying self‐determination theory towards motivational factors of physical activity in people undergoing haemodialyses: A qualitative interview study
Source: Health Expect. 2023 Apr 4;26(3):1368–79. doi: 10.1111/hex.13757 (PMC10154841; doi:10.1111/hex.13757)
Supplement: Supplementary file 1 — Supplementary Table 1. Interview questions for hemodialysis participants. [file HEX-26-1368-s001.docx]

Table S1: Interview questions for hemodialysis participants.

| Interview questions |
| --- |
| 1. How do you view physical activity? Do you think you are fit for physical activity? 2. Are you currently doing physical activity? How do you feel? 3. How do you usually do physical activity? 4. Do you feel capable of engaging in an active physical activity lifestyle? 5. Did you experience support from other people? 6. What motivates you to be physically active? What are the benefits you feel from physical activity? 7. What are your reasons for not engaging in physical activity? What's holding you back from physical activity? 8. How has participating in physical activity changed you? 9. Do you receive any information about physical activity？ |
